# Severe Treatment-Resistant Methemoglobinemia of Unknown Etiology With Recurrence

**DOI:** 10.1155/crcc/5740399

**Published:** 2025-06-17

**Authors:** James Ainsworth, Suresh Pillai

**Affiliations:** Intensive Care Unit, Morriston Hospital, Swansea, UK

**Keywords:** cyanosis, intensive care, methemoglobin, methemoglobinemia, methylene blue

## Abstract

**Introduction:** Methamoglobinemia is a condition characterized by impaired oxygen delivery to the tissues due to the formation of methemoglobin (Met-Hb). Diagnosis is established when Met-Hb levels exceed 5%. Levels over 30% are associated with severe symptoms and can be life-threatening. Pulse oximetry may provide misleading readings, often reporting oxygen saturation around 85%, irrespective of actual oxygenation levels. Management involves discontinuing offending agents, providing supportive care, and use of specific treatment agents. Most commonly, cases report a quick and effective recovery following treatment.

**Case Presentation:** We describe a case of a patient requiring four separate admissions over a 9-month period, all for methemoglobinemia, where the cause remained uncertain. A 25-year-old female was admitted to the intensive care unit (ICU) with sudden onset shortness of breath and cyanosis, and Met-Hb level 55.5%, suspected due to dapsone. She was treated with 1 mg/kg of methylene blue intravenously. Met-Hb levels remained persistently above 20% for a number of days, and reaccumulated following initial decline. Repeated doses of methylene blue were required, and activated charcoal was given. She was readmitted 3 days later, with reaccumulation of Met-Hb. This was again thought secondary to dapsone, given the long half-life of dapsone and potential for enterohepatic recirculation. She had a third admission with methemoglobinemia almost 2 months after the first admission, and again 8 months later. Suggestion was that azathioprine could be the causative agent; however, this has not been reported previously.

**Discussion:** We report a case of a 25-year-old female with repeated admissions with methemoglobinemia. This case highlights the potential for refractory methemoglobinemia requiring repeated treatment, and the importance of longer periods of close observation. It is important to consider the diagnosis in patients without a clear trigger for methemoglobinemia, as an identifiable cause may not always be clear.

## 1. Introduction

Methemoglobinemia is a condition characterized by impaired oxygen delivery to the tissues due to the formation of methemoglobin (Met-Hb), leading to a reduced ability to bind oxygen. This is due to the oxidization of the heme iron in the normal hemoglobin molecule of red blood cells, causing it to change from a ferrous (Fe2^+^) to a ferric (Fe3^+^) state. This results in the formation of Met-Hb. In addition to reducing the ability to bind oxygen, there is a left shift of the hemoglobin–oxygen dissociation curve, further reducing oxygen delivery and exacerbating tissue hypoxia [1].

Methemoglobinemia can be classified into congenital and acquired forms. Congenital cases are often caused by genetic abnormalities and may remain asymptomatic, causing cyanosis or severe complications in neonates [1]. In contrast, acquired methemoglobinemia is typically more acute and can be life-threatening, particularly when hypoxia persists despite supplemental oxygen [1]. Acquired methemoglobinemia can result from exposure to various substances, including medications like local anesthetics (e.g., prilocaine), antimalarials (e.g., primaquine), and certain antibiotics such as dapsone, which is frequently implicated as the most common cause of acquired methemoglobinemia [1, 2]. Other triggers can include household products and chemicals, which further highlight the need for prompt recognition and diagnosis in clinical practice [2].

The clinical presentation of methemoglobinemia is generally with symptoms of hypoxia and cyanosis, and it should be considered in patients with cyanosis despite supplemental oxygen, particularly in patients with no signs of structural heart or lung disease [3, 4]. Workup should include screening for other causes of hypoxia, and prompt testing of Met-Hb levels. In neonates, an absence of structural congenital heart disease should raise consideration for methemoglobinemia. Diagnosis is established when Met-Hb levels exceed 5%, with symptomatic individuals generally presenting with levels above 10%. Levels over 30%–40% are associated with severe symptoms and can be life-threatening, with levels over 70% causing severe hyperemia and death [1]. Caudill et al. [5] describe a case report of severe methemoglobinemia and Met-Hb level of 81.5%, presenting in a coma with fixed dilated pupils, but making a full recovery despite the critical presentation. Notably, pulse oximetry may provide misleading readings in these patients, often reporting oxygen saturation around 85%, irrespective of actual oxygenation levels, as Met-Hb absorbs light at both oxygenated and deoxygenated hemoglobin wavelengths [1, 6].

Management of methemoglobinemia involves discontinuing any offending agents, providing supportive care, and use of specific treatment agents. First-line treatment typically includes intravenous methylene blue, while vitamin C (ascorbic acid) is utilized in cases involving glucose-6-phosphate dehydrogenase (G6PD) deficiency [1, 6, 7]. In severe cases, exchange transfusion and hyperbaric oxygen therapy may be considered [3]. Understanding the pathophysiology, clinical manifestations, and therapeutic approaches to methemoglobinemia is essential for improving patient outcomes.

Most commonly, cases seem to report a quick and effective recovery following starting treatment with a single dose of methylene blue, without the need for repeated dosing [8]. Many case reports, however, demonstrate a variable response to treatment with methylene blue, sometimes requiring multiple doses of treatment, suggesting that it is not possible to accurately predict the response to a single dose of standard therapy and the reduction in Met-Hb levels that will be achieved [2, 3, 6, 9, 10]. Caution and close monitoring should therefore be advised after timely diagnosis and initiation of initial treatment.

## 2. Case Presentation

A 25 year old, Caucasian female with a past medical history of cutaneous vasculitis, under the care of dermatology, atopic dermatitis, and had previously had a history of Graves' disease with total thyroidectomy. There was no family history to note. Prior to admission, she was taking prednisolone 5 mg per day (on long-term steroids for her vasculitis due to difficulty in weaning), levothyroxine, famotidine, alendronic acid, ramipril, folic acid, diphenhydramine hydrochloride, and omeprazole. There was also a history of dapsone use which she had been taking prior to admission, with a recent increase in dose 3 weeks ago to 100 mg for cutaneous vasculitis, which was thought to be the cause of the first presentation with methemoglobinemia in this case.

She was admitted to hospital with sudden onset shortness of breath, headache, and dizziness. She denied any cough or chest pain. On assessment in the emergency department, she was tachypneic and tachycardic, appeared pale and cyanosed, and was commenced on 15 L oxygen via nonrebreather mask. Despite this, pulse oximetry still demonstrated oxygen saturations of 85%. Initial arterial blood gas investigations demonstrated a raised partial pressure of oxygen (PaO_2_) of 38.8 kPa (10–13.3 kPa); despite low oxygen saturations recorded, a partial pressure of carbon dioxide (PaCO_2_) of 3.4 kPa (4.67–6 kPa), a raised lactate of 4 (< 2), bicarbonate 23.2 (22–26), base excess of −1.8 (−2 to +2), and a Met-Hb level of 55.5% (< 1.5%). Blood pressure remained stable throughout. Chest X-ray was normal, and an ECG showed sinus tachycardia. Inflammatory markers were not raised, with a CRP of less than 5. Blood film demonstrated evidence of hemolysis and reticulocytes. Hemoglobin level trended down during admission with a rising bilirubin. There was no evidence of G6PD deficiency on testing. Discussion with hematology suggested this was likely related to dapsone, and hemoglobin levels improved later during admission, following stopping the dapsone and treatment for the methemoglobinemia. Delayed hemolytic anemia has been reported in other cases of dapsone-related methemoglobinemia with high Met-Hb levels [11].

Following admission to the emergency department, the decision was made to admit the patient to the intensive care unit (ICU) for high-flow nasal oxygen (HFNO) and for an arterial line to enable regular blood gas monitoring, given the lack of accuracy of the pulse oximeter. The team liaised with the poisons service, and she was treated with 1 mg/kg of methylene blue intravenously. Met-Hb levels remained persistently raised above 20% however. There was an initial decline in Met-Hb levels from 55.5% to 11.1% over the first 10 h, which then reaccumulated following treatment over the next 8 h to over 20%. The patient required repeated doses of treatment with methylene blue, receiving a total of 4 mg/kg. Further discussions with the poison center suggested potential risks with repeated doses of methylene blue; therefore, advising to give activated charcoal and to check Met-Hb levels every 2 h. A nasogastric (NG) tube was inserted and the patient was given enteral activated charcoal via the NG route for comfort, receiving 50 mg four times daily for 48 h. The poison center advised if Met-Hb levels rose above 30% then to restart methylene blue as an infusion of 0.1 mg/kg, and then to stop if decreasing below 20%. Another treatment option suggested was exchange transfusion, but it was thought this could have an impact on future vasculitis treatment. Thankfully, levels did not rise further, and further methylene blue was not required. The Met-Hb levels were still slow to shift, however, with levels remaining above 20% for over 2 days, with then a slow decline down without further treatment, remaining on the ICU for ongoing monitoring. The patient was admitted to the ICU for a total of 5 days. Met-Hb levels reduced to 8.8% by discharge from the ICU. Oxygen saturations were still 84% on room air, but with an adequate partial pressure of oxygen of 9.9 kPa. She was later discharged from hospital with a plan for medical team follow-up and repeat bloods in the next few days. Her dose of prednisone was increased to 30 mg per day.

She was then readmitted just 3 days later following discharge, with a reaccumulation of Met-Hb. Presentation was similar, with a Met-Hb level of 35.1%. She was again admitted to the ICU and received treatment with intravenous methylene blue, as per advice from the national poison center. This appeared to be more effective during this second admission, with Met-Hb levels reducing to 7.3%. There was again a slow rise following initial treatment, but she did not require any further treatment, with Met-Hb levels and symptoms resolving. She was then discharged the following day, requiring only a 2-day admission on this occasion. This was again thought secondary to previous dapsone use, given the long half-life of dapsone of over 30 h and potential for enterohepatic recirculation, with the potential for reaccumulation in Met-Hb [1].

Interestingly, she had a further admission with methemoglobinemia almost 2 months after the first admission. Presentation was with an acute history of 2 days of increased shortness of breath, fatigue, and headache, and she was cyanotic on presentation to the emergency department. Met-Hb level on this admission was 32.2%, reducing to 10% with methylene blue treatment. She did not require ICU admission during this admission. The cause of methemoglobinemia on this occasion, however, was less clear, having stopped dapsone for some time (prior to the first admission). She had been started on azathioprine for vasculitis following initial discharge but denied any dapsone use or any other illicit substances. Discussions with the poison center felt dapsone was an unlikely cause, stating that dapsone does not cause this presentation 6 weeks after ingestion. Suggestion was that azathioprine could be the causative agent; however, this has not been reported previously.

A few months later (8 months after the first admission noted here) she was again admitted with methemoglobinemia. See Table 1 which displays all four admissions with methemoglobinemia. This time she had recently returned from a trip to Africa where she contracted malaria, receiving treatment with intravenous artesunate, as well as antibiotics for suspected pyelonephritis. Following admission, she was found to have a Met-Hb level of 45% and was again admitted to the ICU. She received a single dose of methylene blue and levels declined to 8.1% over 48 h. The question of the cause may be uncertain across admissions, particularly given the repeated admissions.

## 3. Discussion

This case report is aimed at contributing to the existing body of literature by detailing a recent case of methemoglobinemia. This case again emphasizes the importance of rapid identification and intervention in affected patients, but with some key learning points. We report a case of a 25-year-old female with repeated separate admissions with methemoglobinemia over a 9-month period, the first of which required multiple doses of treatment and repeated discussions with the poison center, with a longer ICU length of stay and hospital admission. Despite many case reports showing a rapid response and decline in Met-Hb levels following initial treatment with methylene blue, this case shows the potential for treatment-resistant and refractory methemoglobinemia, highlighting the variable response to standard treatment with methylene blue in some patients. Noting this, it is important to consider longer periods of close observation even if an initial decline in Met-Hb levels is seen following the first round of treatment. The cause for methemoglobinemia may not always be clear, so the diagnosis should be considered in typical cases and screened for, even if there is no obvious or known trigger for methemoglobinemia noted in the patient's history.

## Figures and Tables

**Table 1:** Outline of the recurrent admissions.

**Admission**	**Clinical summary**	**Key medications at time of admission**	**Suspected cause**
First	Severe cyanosis and hypoxia despite high-flow oxygen. Met-Hb 55.5%. Required ICU admission, multiple doses of methylene blue, activated charcoal via NG.	Dapsone (recently increased), prednisolone, levothyroxine, diphenhydramine	Suspected dapsone-induced
Second	Representation 3 days postdischarge. Met-Hb 35.1%. ICU admission, improved with methylene blue.	No new medications; residual dapsone exposure likely	Delayed dapsone clearance
Third	Around 2 months after first admission. Met-Hb 32.2%. Treated with methylene blue, no ICU required.	Azathioprine newly initiated; no recent dapsone use	Possibly azathioprine (unreported)
Fourth	Approximately 8 months after first admission. Posttravel illness with malaria (treated with IV artesunate). Met-Hb 45%. ICU admission, improved after one dose of methylene blue.	Artesunate, antibiotics (for pyelonephritis), background immunosuppression	Unclear; possibly artesunate-related

## Data Availability

Data sharing is not applicable to this article, as no new data were created or analyzed in this study.
