# Supplementary material for: Severe Treatment-Resistant Methemoglobinemia of Unknown Etiology With Recurrence
Source: Case Rep Crit Care. 2025 Jun 17;2025:5740399. doi: 10.1155/crcc/5740399 (PMC12187437; doi:10.1155/crcc/5740399)
Supplement: Supporting Information — Additional supporting information can be found online in the Supporting Information section. [file 5740399.f1.pdf]

CONSENT FORM FOR CASE REPORTS[1]

For a patient's consent to publication of information about them in a journal or thesis

Name of person described in article or shown ~~in photograph~~: CHLOE JONES

Subject matter of photograph or article: HISTORY OF METHAEMOGLOBINAEMIA + ICU ADMISSION, BLOOD RESULTS ETC.

Title of article: ______________________________

Medical practitioner or corresponding author: JAMES AINSWORTH

I CHLOE JONES [insert full name] give my consent for this information about MYSELF OR MY CHILD OR WARD/MY RELATIVE [insert full name]:______________________, relating to the subject matter above ("the Information") to appear in a journal article, or to be used for the purpose of a thesis or presentation.

I understand the following:

1. The Information will be published without my name/child's name/relatives name attached and every attempt will be made to ensure anonymity. I understand, however, that complete anonymity cannot be guaranteed. It is possible that somebody somewhere - perhaps, for example, somebody who looked after me/my child/relative, if I was in hospital, or a relative - may identify me.
2. The Information may be published in a journal which is read worldwide or an online journal. Journals are aimed mainly at health care professionals but may be seen by many non-doctors, including journalists.
3. The Information may be placed on a website.
4. I can withdraw my consent at any time before online publication, but once the Information has been committed to publication it will not be possible to withdraw the consent.

Signed: Jones Date: 22.12.23

Signature of requesting medical practitioner/health care worker:

[signature] Date: 22/12/23

---

[1] Adapted from *BMJ Case Reports* consent form.
Division Research Development and Support, Faculty of Health Sciences, Stellenbosch University, South Africa. Consent form for case reports. Version1. Sept 2008.
